# Pancreatic Parenchymal Atrophy and Pancreatic Fat Accumulation Measured by Multidetector Computed Tomography as a Stable Marker of Chronic Progressive Type 2 Diabetes Mellitus—A Cross Sectional Observational Study

**DOI:** 10.1055/s-0044-1779667

**Published:** 2024-02-27

**Authors:** Kshipra Devadiga, Khanak K Nandolia, Mahendra Singh, Pankaj Sharma, Udit Chauhan, Ravi Kant

**Affiliations:** 1 Department of Diagnostic and Intervention Radiology, All India Institute of Medical Sciences, Rishikesh, Uttarakhand, India; 2 Department of Internal Medicine, All India Institute of Medical Sciences, Rishikesh, Uttarakhand, India; 3 Department of Community and Family Medicine, All India Institute of Medical Sciences, Rishikesh, Uttarakhand, India

**Keywords:** type 2 diabetes mellitus, pancreatic volume, pancreatic fat, multidetector computed tomography

## Abstract

**Background** The most crucial step in the management of type 2 diabetes is identifying its pathogenesis and progression. Fat accumulation in the pancreas and decreased parenchymal volume can influence pancreatic function due to insulin resistance or β-cell dysfunction. This study aims to find out the difference in pancreatic volume and fat content by using contrast-enhanced computed tomography (CECT) between normal subjects and patients with different durations of type 2 diabetes mellitus (T2DM).

**Methods** This was a cross-sectional study. Patients who underwent CECT abdomen for the evaluation of conditions other than pancreatic origin were included. The study group was divided into three subgroups according to the duration of diabetes as <5 years, 5 to 10 years, and >10 years. In total, 40 nondiabetic controls were included. Pancreatic fat volume and parenchymal volume were measured in cm ^3^ using CECT. Correlation between pancreatic parenchymal and fat volume with the duration of T2DM as well as with levels of hemoglobin A1c, random blood sugar, serum triglyceride, low-density lipoproteins, and high-density lipoproteins was done.

**Results** T2DM patients had significantly ( *p* < 0.001) lower pancreatic parenchymal volume (mean value of 57.08 ± 8.26 cm ^3^ in diabetics and 72.23 ± 3.41 cm ^3^ in controls) and higher pancreatic fat volume (mean value of 3.08 ± 1.90 cm ^3^ in diabetics and 0.67 ± 0.27cm ^3^ in controls) as compared to nondiabetic controls. In patients with T2DM, as the duration of T2DM increased, pancreatic parenchymal volume decreased and pancreatic fat volume increased.

**Conclusion** Reduction in pancreatic volume and fat deposition may have a role in the onset and progression of diabetes. Determining the pancreatic volume and fat content would be useful for identifying high-risk patients and determining the pathogenesis of the development of diabetes.

## Introduction

Diabetes is an “iceberg” disease. One of the most prevalent comorbidities in the modern world is type 2 diabetes mellitus (T2DM). It requires lifelong monitoring due to its high morbidity. The burden of diabetes is significant and growing both globally and in developing countries like India, as a result of increased incidence of obesity and unhealthy lifestyles. In India, there are currently believed to be 77 million diabetics, and by 2045, there are expected to be over 134 million. More than 57% of these individuals are still undiagnosed. 1

Untreated diabetic patients experience a variety of chronic complications leading to morbidity and mortality. Diabetics have a higher incidence of cardiovascular and cerebrovascular ischemic events. Microvascular complications, such as retinopathy, neuropathy, and nephropathy, lower the quality of life and increase the risk of early death. 1 Diabetes patients have a 10 times higher risk of having a lower limb amputation than nondiabetics. 3 Therefore, the most crucial step in the management of T2DM is identifying its presence and progression.

Excess dietary lipids accumulate in the subcutaneous fat initially. Further, an increase in blood lipid content causes its ectopic accumulation in pancreas. Lipid accumulation in the pancreas may contribute to the decline in β-cell activity. As a result of oxidative stress and endoplasmic reticulum stress brought on by high-fat diets, β-cell apoptosis occurs. 2 3 β-cell failure is also caused by defects in protective mechanisms, such as a reduction in inducible nitric oxide synthase or mitochondrial β-oxidation caused by glucolipotoxicity. 4 5 These mechanisms cause a reduction in pancreatic volume including beta cells.

Our primary objective was to correlate pancreatic parenchymal volume and fat content with the duration of T2DM. Secondary objective was to determine the progression of T2DM by measuring the pancreatic fat and parenchymal volume using multidetector computed tomography (MDCT).

## Materials and Methods

A cross-sectional observational study was conducted over a period of 18 months from July 2021 to January 2023, at the Department of Diagnostic and Interventional Radiology, in association with the Department of Internal Medicine. The study group had 191 patients and the control group had 40 patients. Patients were recruited on the basis of inclusion and exclusion criteria after taking informed consent.

### Inclusion Criteria for the Study Group

Inclusion criteria for the study group were as follows.

1. Adult patients (>18 years) with T2DM and undergoing contrast-enhanced computed tomography (CECT) abdomen for other indications.
2. Patients willing to give an informed written consent.

### Inclusion Criteria for the Control Group

Inclusion criteria for the control group were as follows.

1. Adult nondiabetic patients underwent CECT abdomen for other indications.
2. Patients willing to give an informed written consent.

### Exclusion Criteria for Both Study and Control Groups

Exclusion criteria for both groups were as follows.

1. Patients with history/clinical diagnosis of acute pancreatitis, chronic pancreatitis, or pancreatic malignancy.
2. Any contraindication for intravenous iodinated contrast administration.
3. Any medical/surgical intervention that could affect pancreatic volume.

A thorough clinical history was obtained, and demographics were recorded. The duration of T2DM after detection, patient's body weight and height, levels of fasting and postprandial blood glucose, hemoglobin (Hb)A1C, serum triglycerides, and serum high- and low-density lipoproteins were recorded from patient's medical records. Images were acquired in 128 slice dual source CT scanner (Somatom Definition Flash, Siemens Healthcare, Forchheim, Germany). The following scan acquisition parameters were used: 120 kV, 200 mAs, and 3 mm slice thickness. Axial unenhanced images of the abdomen were acquired. Contrast-enhanced images were acquired after an intravenous injection of low osmolality (350 mg of iodine/dL) iodinated contrast agent at a rate of 3 to 4 mL/s at the dose of 1 to 2 mL/kg body weight. Portal venous phase axial images were evaluated for pancreatic parenchymal volume and fat content. CECT images could identify the normal pancreatic parenchyma and vascular structures correctly. The pancreatic parenchymal boundaries were drawn using the free selection tool included in the Syngo.via workstation (Siemens Healthcare, Forchheim, Germany). The volumetry was used to measure the volume of the selected region in each slice, encompassing the entire pancreatic parenchyma in the slice while excluding adjacent organs and vessels.

**Fig. 1:**
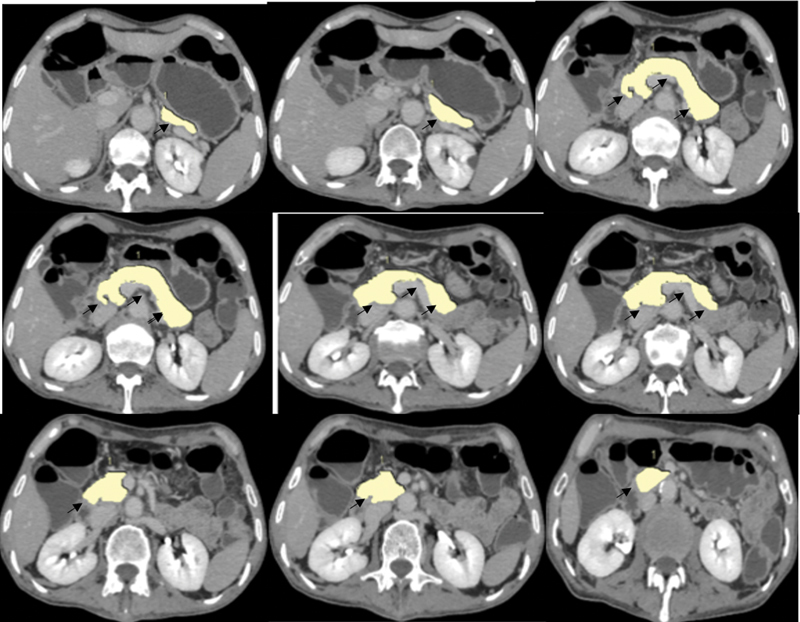
MDCT axial images for pancreatic volume determination. Pancreatic parenchyma volume was measured using a semiautomatic segmentation technique with a dedicated computer 3D workstation. Segmentation of the pancreas was achieved by isolating pancreatic parenchyma ( *short black arrows* ) from the upper body to the lower head part. The pancreas volume ( *yellow color* ) was segmented from the adjacent tissues by manual trace in few slices. Segmentation software automatically performed interpolating between the user-defined traces.

The pancreatic parenchyma was manually traced in all slices with a slice interval of 1 mm, and the volume and fat of the pancreas were then determined by the summation of the segmented pancreatic area. Using the above-mentioned techniques, the pancreatic volume was initially determined as demonstrated in Fig. 1 . All manual trace measurements were performed by a senior radiologist trained in gastrointestinal and pancreatic imaging and 10 years of experience in abdominal imaging. Intraobserver variations were analyzed and found to have good reliability (94% agreement, intraclass correlation coefficient 0.92, 95% confidence interval 0.91–0.94). The volume of the pancreas where the HU < 0 was defined as PFV and HU > 0 was defined as PPV. Care was taken not to include the peripheral margin of the pancreas to avoid any influence of the partial volume effect and vascular and other adjacent structures.

## Statistical Analysis

SPSS—Statistical Package for Social Sciences version 24 (IBM, New York, United States) was used for all calculations. Excel spreadsheet (Microsoft, Washington, United States) was used for data entry and data organization. Every effort was made to ensure that there were correct data entry.

Frequency was used to describe categorical variables. Continuous variables were described as mean, standard deviation, or median with appropriate interquartile range. The means in the two groups were compared using the student *t* -test and Mann–Whitney U test as applicable. Analysis of variance was used to compare means in more than two groups. Regression analyses were used to document independent predictors of the desired outcome. Statistical significance was kept at *p* < 0.05.

## Results

A total of 191 patients were included with 151 patients in the diabetic group and 40 patients in the nondiabetic control group. No patients were dropped out because of missing data or were lost to follow-up.

In the diabetic group, 85 (56.3%) male patients and 66 (43.7%) female patients were included.

Association between the T2DM and parameters like pancreatic fat, parenchymal volume, body mass index (BMI), and random blood sugar (RBS) are summarized in Table 1 .

**Table 1:** Correlation of T2DM with demographics, BMI, pancreatic parenchymal and fat volume, RBS

Parameters			*p* -Value
	Case (diabetics) ( *n* = 151)	Controls ( *n* = 40)	
Age (years) e	53.26 ± 5.26	49.73 ± 7.10	0.002 a
Age e			0.002 b
31–40 y	4 (2.6%)	6 (15.0%)	
41–50 y	39 (25.8%)	16 (40.0%)	
51–60 y	99 (65.6%)	15 (37.5%)	
61–70 y	9 (6.0%)	3 (7.5%)	
Gender			0.668 c
Male	85 (56.3%)	21 (52.5%)	
Female	66 (43.7%)	19 (47.5%)	
BMI (kg/ m ^2^ ) e	25.70 ± 1.79	22.51 ± 1.35	<0.001 d
BMI e			<0.001 b
18.5–22.9 kg/m ^2^	8 (5.3%)	25 (62.5%)	
23.0–24.9 kg/m ^2^	43 (28.5%)	15 (37.5%)	
25.0–29.9 kg/m ^2^	99 (65.6%)	0 (0.0%)	
30.0–34.9 kg/m ^2^	1 (0.7%)	0 (0.0%)	
Pancreatic volume (cm ^3^ ) e	57.08 ± 8.26	72.23 ± 3.41	<0.001 d
Pancreatic fat (cm ^3^ ) e	3.08 ± 1.90	0.67 ± 0.27	<0.001 a
RBS (mg/dL) e	135.01 ± 9.03	101.65 ± 12.13	<0.001 d

Abbreviations: BMI, body mass index; DM, diabetes mellitus; HDL, high-density lipoproteins; LDL, low-density lipoproteins; RBS, random blood sugar.

a Wilcoxon–Mann–Whitney U test.

b Fisher's exact test.

c Chi-squared test.

d *t* -test.

e Significant at *p* < 0.05.

**Fig. 2:**
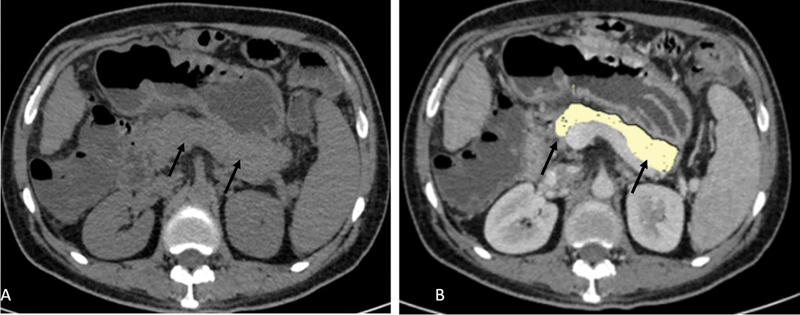
A 49-year-old male nondiabetic patient underwent CECT thorax abdomen for the evaluation of disseminated tuberculosis. Noncontrast axial image **(A)** and postcontrast portal-venous phase axial image **(B)** show pancreatic parenchyma ( *short black arrows* ), outlined by manual tracing. Parenchymal volume was 70.72cc and fat volume was 0.26cc in this case.

Mean pancreatic parenchymal volume was significantly higher in controls (72.23 ± 3.41 cm ^3^ ) when compared to patients with T2DM (57.08 ± 8.26 cm ^3^ ). Fig. 2 shows pancreatic parenchymal and fat volume measurements in a nondiabetic individual ( Fig. 2 ).

**Fig. 3:**
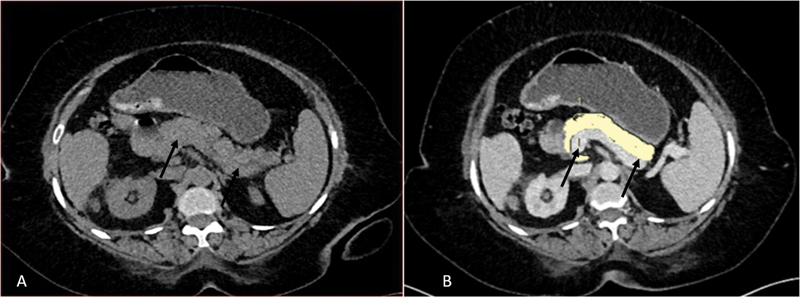
A 50-year-old female patient with a history of diabetes for 4 years was diagnosed with carcinoma breast. She underwent CECT of the thorax and abdomen as a part of metastatic workup. Noncontrast **(A)** and postcontrast portal-venous phase **(B)** axial images show pancreatic parenchyma ( *black arrows* ) outlined by manual tracing. Pancreatic parenchymal volume was 65.34cc and fat volume was 1.82cc in this case.

**Fig. 4:**
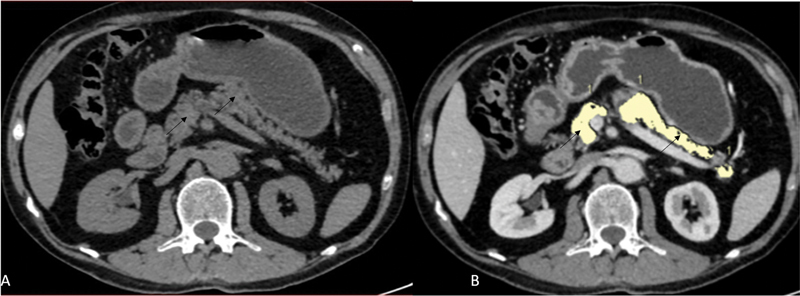
A 64-year-old male patient with a history of diabetes for 9.5 years underwent CECT abdomen for the evaluation of suspected colonic carcinoma. Noncontrast **(A)** and postcontrast portal-venous phase **(B)** axial images show pancreatic parenchyma ( *black arrows* ) outlined by manual tracing. Pancreatic parenchymal volume was 48.66cc, and fat volume was 5.34cc in this case.

**Fig. 5:**
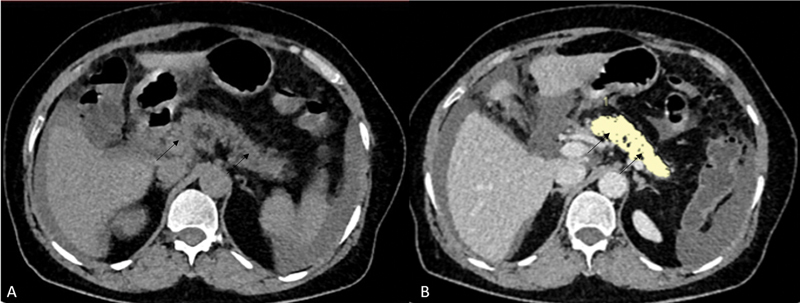
A 56-year-old female patient with a history of diabetes for 15 years underwent CECT of the abdomen and pelvis for evaluation of an adnexal mass. Noncontrast **(A)** and postcontrast portal-venous phase **(B)** axial images show pancreatic parenchyma ( *black arrows* ) outlined by manual tracing. On analysis pancreatic parenchymal volume came out to be 39.30cc and fat volume 8.88cc.

The correlation between the duration of T2DM and other parameters are summarized in Table 2 . As the duration of T2DM progresses, mean pancreatic volume decreases and mean pancreatic fat increases. Mean pancreatic volume and pancreatic fat in patients with T2DM with duration <5 years, 5 to 10 years, and > 10 years were 64.22 ± 6.02 cm ^3^ , 57.67 ± 5.21 cm ^3^ , 49.21 ± 5.29 cm ^3^ and 1.25 ± 0.61 cm ^3^ , 3.04 ± 1.17 cm ^3^ , 4.99 ± 1.46 cm ^3^ , respectively. Figs. 3 , 4 , and 5 show measurements of pancreatic parenchymal and fat volumes with the duration of T2DM for 4, 9.5, and 15 years respectively.

**Table 2:** Correlation of T2DM duration with demographics, BMI, pancreatic parenchymal and fat volume, and various biochemical parameter values

Parameters	Duration of T2DM (mean ± standard deviation)			*p* -Value
	<5 y ( *n* = 51)	5–10 y ( *n* = 50)	10–15 y ( *n* = 50)	
Age (years) e	48.43 ± 4.64	54.12 ± 3.43	57.32 ± 3.00	<0.001 a
Age e				<0.001 b
31–40 y	4 (7.8%)	0 (0.0%)	0 (0.0%)	
41–50 y	31 (60.8%)	7 (14.0%)	1 (2.0%)	
51–60 y	15 (29.4%)	41 (82.0%)	43 (86.0%)	
61–70 y	1 (2.0%)	2 (4.0%)	6 (12.0%)	
Gender				0.182 c
Male	34 (66.7%)	26 (52.0%)	25 (50.0%)	
Female	17 (33.3%)	24 (48.0%)	25 (50.0%)	
BMI (kg/ m ^2^ ) e	24.36 ± 1.36	26.47 ± 1.71	26.31 ± 1.46	<0.001 d
BMI e				<0.001 b
18.5–22.9 kg/m ^2^	8 (15.7%)	0 (0.0%)	0 (0.0%)	
23.0–24.9 kg/m ^2^	25 (49.0%)	11 (22.0%)	7 (14.0%)	
25.0–29.9 kg/m ^2^	18 (35.3%)	39 (78.0%)	42 (84.0%)	
30.0–34.9 kg/m ^2^	0 (0.0%)	0 (0.0%)	1 (2.0%)	
Duration of T2DM (years) e	2.49 ± 1.36	7.00 ± 1.33	12.35 ± 1.35	<0.001 a
Pancreatic Volume (cm ^3^ ) e	64.22 ± 6.02	57.67 ± 5.21	49.21 ± 5.29	<0.001 d
Pancreatic fat (cm ^3^ ) e	1.25 ± 0.61	3.04 ± 1.17	4.99 ± 1.46	<0.001 d
RBS (mg/dL)	137.06 ± 11.22	134.64 ± 7.56	133.28 ± 7.51	0.317 a
HbA1C (%)	7.67 ± 1.21	7.79 ± 0.83	7.61 ± 1.28	0.379 a
S. triglyceride (mg/dL)	182.51 ± 20.98	185.40 ± 18.12	179.30 ± 20.17	0.148 a
LDL (mg/dL)	148.20 ± 25.39	141.00 ± 18.54	145.50 ± 21.86	0.308 a
HDL cholesterol (mg/dL) e	45.73 ± 7.78	45.20 ± 5.34	41.08 ± 5.79	0.001 a

Abbreviations: BMI, body mass index; DM, diabetes mellitus; HDL, high-density lipoproteins; LDL, low-density lipoproteins; RBS, random blood sugar.

a Kruskal–Wallis test.

b Fisher's exact test.

c Chi-squared test.

d One-way ANOVA.

e Significant at *p* < 0.05.

The correlation between pancreatic parenchymal volume and other parameters are summarized in Table 3 . There was a significant negative correlation between pancreatic volume with the duration of diabetes mellitus (rho = − 0.89, *p* < 0.001), pancreatic fat (rho = − 0.93, *p* < 0.001), BMI (rho = − 0.35, *p* < 0.001), and serum triglyceride levels (rho = − 0.21, *p* < 0.001). There was a significant positive correlation between pancreatic volume with serum high-density lipoproteins (HDL) cholesterol levels (rho = 0.16, *p* < 0.046). There was no significant correlation between pancreatic volume with random blood sugar, HbA1c, and serum low-density lipoproteins (LDL) cholesterol levels.

**Table 3:** Correlation of pancreatic parenchymal volume with demographics, BMI, pancreatic parenchymal and fat volume, and various biochemical parameter values

Parameters	Pancreatic volume in cm ^3^ (mean ± standard deviation)	*p* -Value
Age (years) e	Correlation coefficient (rho) = − 0.82	<0.001 a
Age e		<0.001 b
31–40 y	69.72 ± 7.39	
41–50 y	64.85 ± 5.23	
51–60 y	54.79 ± 5.97	
61–70 y	42.97 ± 5.44	
Gender		0.534 c
Male	57.44 ± 8.76	
Female	56.61 ± 7.60	
BMI (kg/m ^2^ ) e	Correlation coefficient (r) = − 0.35	<0.001 d
BMI e		<0.001 b
18.5–22.9 kg/m ^2^	67.22 ± 3.47	
23.0–24.9 kg/m ^2^	59.58 ± 8.87	
25.0–29.9 kg/m ^2^	55.20 ± 7.42	
30.0–34.9 kg/m ^2^	55.00 ± 0	
Duration of DM (years) e	Correlation coefficient (rho) = − 0.89	<0.001 a
Pancreatic fat (cm ^3^ ) e	Correlation coefficient (rho) = − 0.93	<0.001 a
RBS (mg/dL)	Correlation coefficient ( *r* ) = 0.08	0.315 d
HbA1C (%)	Correlation coefficient (rho) = 0.01	0.915 a
S. triglyceride (mg/dL) e	Correlation coefficient (rho) = − 0.21	0.010 a
LDL (mg/dL)	Correlation coefficient ( *r* ) = 0.04	0.587 d
HDL cholesterol (mg/dL) ^e^	Correlation coefficient ( *r* ) = 0.16	0.046 d

Abbreviations: BMI, body mass index; DM, diabetes mellitus; HDL, high-density lipoproteins; LDL, low-density lipoproteins; RBS, random blood sugar.

a Spearman correlation.

b Kruskal–Wallis test.

c *t* -test.

d Pearson's correlation.

e Significant at *p* < 0.05.

The correlation between pancreatic fat and other parameters is summarized in Table 4 .

**Table 4:** Correlation of pancreatic fat volume with demographics, BMI, pancreatic parenchymal and fat volume, and various biochemical parameter values

Parameters		Pancreatic fat in cm ^3^ (mean ± standard deviation)	*p* -Value
Age (years) d		Correlation coefficient (rho) = 0.79	<0.001 a
Age d			<0.001 b
	31–40 y	0.81 ± 0.47	
	41–50 y	1.36 ± 0.93	
	51–60 y	3.58 ± 1.54	
	61–70 y	6.04 ± 2.16	
Gender			0.381 c
Male		3.00 ± 1.98	
Female		3.19 ± 1.81	
BMI (kg/ m ^2^ ) d		Correlation coefficient (rho) = 0.42	<0.001 a
BMI d			<0.001 b
18.5–22.9 kg/m ^2^		0.78 ± 0.29	
23.0–24.9 kg/m ^2^		2.44 ± 1.82	
25.0–29.9 kg/m ^2^		3.55 ± 1.81	
30.0–34.9 kg/m ^2^		3.37 ± 0	
Duration of DM (y) d		Correlation coefficient (rho) = 0.93	<0.001 a
Pancreatic volume (cm ^3^ ) d		Correlation coefficient (rho) = − 0.93	<0.001 a
RBS (mg/dL)		Correlation coefficient (rho) = − 0.08	0.306 a
HbA1C (%)		Correlation coefficient (rho) = 0.01	0.894 a
S. Triglyceride (mg/dL) d		Correlation coefficient (rho) = 0.18	0.028 a
LDL (mg/dL)		Correlation coefficient (rho) = − 0.04	0.653 a
HDL cholesterol (mg/dL)		Correlation coefficient (rho) = − 0.16	0.055 a

Abbreviations: BMI, body mass index; DM, diabetes mellitus; HDL, high-density lipoproteins; LDL, low-density lipoproteins; RBS, random blood sugar.

a Spearman correlation.

b Kruskal–Wallis test.

c Wilcoxon–Mann–Whitney U test.

d Significant at *p* < 0.05.

There was a significant positive correlation between pancreatic fat volume and duration of T2DM (rho = 0.93, *p* < 0.001), BMI (rho = 0.42, *p* < 0.001), and serum triglyceride levels (rho = 0.18, *p* < 0.001). There was a significant negative correlation between pancreatic fat volume and the pancreatic parenchymal volume (rho = −0.93, *p* < 0.001). There was no significant correlation between pancreatic fat volume and RBS, HbA1c, and serum HDL and LDL cholesterol levels.

### Regression Analysis

The duration of T2DM was significantly correlated with pancreatic parenchymal volume and pancreatic fat volume, after controlling the confounding factors like age, gender and BMI.

For 1 year increase in the duration of T2DM, pancreatic parenchymal volume reduced by 1.31 cm ^3^ and pancreatic fat volume increased by 0.39 cm ^3^ .

## Discussion

Current diagnostic criteria for T2DM are based on fasting and postprandial blood glucose levels, and HbA1c levels. 4 Blood glucose levels are point variables that show the status of intake of medications and sugars at that point of time. HbA1c represents blood sugar control over a period of 8 to 12 weeks and is easy to measure and does not require fasting status. However, its value is influenced by various conditions like hemoglobinopathies, vitamin B12 deficiency, decreased erythropoiesis, administration of erythropoietin, iron, reticulocytosis, chronic liver disease, alcoholism, chronic renal failure, decreased erythrocyte pH, splenectomy, etc. 6

These tests represent the blood sugar control which may be variable according patient's compliance with antidiabetic medications. They do not tell us about the status of pancreatic health, which is the main seat of pathology. Furthermore, abnormalities in these blood tests would not appear until 70 to 90% of the beta cells have been destroyed. 7

If the disease progression continues, the patients can no longer be managed by oral hypoglycemic drugs alone and will need to be managed with insulin replacement therapy once there is near complete destruction of beta-cells. Therefore, if the pathogenesis is detected in the early phase, lipid-lowering drugs and antioxidants can be introduced in the management of T2DM to delay the onset and progression of the disease. 8 9 Further pancreatic lipomatosis can cause exocrine deficiency and malabsorption syndromes. Pancreatic parenchymal loss and fatty replacement is largely irreversible and show the true extent of disease progression and its impact over the pancreas. Unlike HbA1C and blood glucose levels, pancreatic fat replacement is not reversed with antidiabetic measures. 10

Therefore, identifying the status of pancreatic parenchyma is important in the management of T2DM. In this study, we found that patients with T2DM had smaller pancreatic volumes and higher pancreatic fat content than normoglycemic subjects. Our study also revealed that in patients with T2DM, as the duration of diabetes increased, the pancreatic volume decreased and the pancreatic fat increased. It resulted in an increased fat percentage in the pancreatic parenchyma.

Previous studies with a small sample size had demonstrated that diabetes patients who were either insulin-dependent or insulin-independent had a lower pancreas volume than healthy controls. 11 12 13 Additionally, it was discovered that patients with type 1 diabetes or young patients with maturity-onset diabetes had smaller pancreatic volumes than controls or T2DM patients. 14 These findings imply that impaired insulin secretion is linked to decreased pancreatic volume.

According to Tushuizen et al, 15 patients with T2DM had more pancreatic fat accumulation than control subjects. Heni et al reported that in patients with impaired glucose metabolism, pancreatic fat content, measured by magnetic resonance spectroscopy, was found to be a significant predictor of decreased insulin secretion than visceral fat levels alone. However, the sample size was small in this study. 16

Sharma et al. 10 found a strong association between pancreatic lipomatosis and T2DM in the Indian population. The duration of the diabetes, however, was not studied, and there was no association with the lipid profile.

Our study proved that, with T2DM progression, there is a significant loss of pancreatic parenchymal volume and an increase in pancreatic fat volume, which can be measured by MDCT.

## Limitations

In this study, we measured total pancreatic parenchymal volume and not the beta cell mass that secretes insulin. No tests were done to assess the beta cell function (IGI) and to evaluate the insulin resistance (HOMA-IR). These tests were not included in the study due to cost constraints.

The study design was cross-sectional without any longitudinal follow-up of the patients. Therefore, we are unable to draw causal conclusions or generalize the present findings to predict the incidence of diabetes. Longitudinal studies with serial evaluations are necessary to determine if pancreatic volumetric or fat content parameters contribute to or are a result of the type 2 diabetes disease process. Pancreatic volume remains on a relatively steady plateau between ages of 20 and 60 years. 17 Hence, we did not analyze the effect of age on pancreatic volume. However, the effect of BMI on pancreatic volume in diabetic patients further needs to be investigated.

## Future Scope

We found out in our study that pancreatic parenchymal volume has negative and pancreatic fat has a positive association with parameters like BMI, and serum triglyceride. In the future, randomized controlled studies can be done with the introduction of hypolipidemic agents in patients with a high risk of diabetes/prediabetes or newly diagnosed diabetic patients.

Similarly, there was a positive association between pancreatic parenchymal volume and HDL cholesterol. Future studies can be done after increasing the blood levels of HDL cholesterol and introducing antioxidants to know whether there is a delay in the development and progression of diabetes in high-risk individuals or prediabetic subjects.

As discussed, the presence of increased pancreatic fat and decreased pancreatic parenchymal volume can be potential surrogate markers for the development and progression of T2D which warrants further investigations.

## Conclusion

According to our research, reduction in pancreatic volume and fat deposition may have a role in the onset and progression of diabetes. In our opinion, determining the pancreatic volume and fat content would be useful for identifying high-risk patients and determining the reason for diabetes development.
